# What to choose and why to use – a critical review on the clinical relevance of rASRM, EFI and Enzian classifications of endometriosis

**DOI:** 10.52054/FVVO.13.4.041

**Published:** 2021-12-30

**Authors:** G Hudelist, L Valentin, E Saridogan, G Condous, M Malzoni, H Roman, D Jurkovic, J Keckstein

**Affiliations:** Department of Gynaecology, Centre for Endometriosis, Hospital St. John of God; Rudolfinerhaus Private Clinic & Campus, Vienna, Austria; Scientific Endometriosis Foundation (Stiftung Endometrioseforschung/ SEF), Westerstede, Germany; Department of Obstetrics and Gynaecology, University of Malmö, Sweden; Department of Clinical Sciences Malmö, Lund University, Sweden; Institute for Women’s Health, University College London, London, UK; Acute Gynaecology, Early Pregnancy and Advanced Endoscopy Surgery Unit, Sydney Medical School Nepean, University of Sydney Nepean Hospital, Australia; Endoscopica Malzoni - Centre for Advanced Endoscopic Gynecological Surgery, Avellino, Italy; Endometriosis Centre, Clinique Tivoli-Ducos, Bordeaux, France; Endometriosis Clinic Dres. Keckstein, Villach, Austria

**Keywords:** Deep endometriosis (DE), classification systems, rASRM, EFI, #Enzian classification

## Abstract

**Background:**

Endometriosis is a common benign gynaecological disease that affects pelvic structures and causes adhesions. Endometriosis outside the pelvis exists but is rarer. Deep endometriosis may affect organs such as the urinary bladder, ureters, bowel and sacral roots. Adenomyosis (growth of endometrium in the myometrium, sometimes explained by disruption of the uterine junctional zone) frequently co-exists with deep endometriosis. Over the past decades, multiple attempts have been made to describe the anatomical extent of endometriosis. Out of approximately 20 classification systems suggested and published so far, three have gained widespread acceptance. These are the rASRM (American Society of Reproductive Medicine) classification, the Endometriosis Fertility Index (EFI) and the Enzian classification. Ideally, a classification system should be useful both for describing disease extent based on surgical findings and results of imaging methods (ultrasound, magnetic resonance imaging).

**Objectives:**

To highlight the advantages and disadvantages of the three classification systems.

**Methods:**

This is a narrative review based on selected publications and experience of the authors. We discuss the current literature on the use of the rASRM, EFI and Enzian classification systems for describing disease extent with imaging methods and for prediction of fertility, surgical complexity, and risk of surgical complications. We underline the need for one universally acceptable terminology to describe the extent of endometriosis.

**Conclusions:**

A useful classification system for endometriosis should describe the sites and extent of the disease, be related to surgical complexity and to disease-associated symptoms, including subfertility and should satisfy needs of both, imaging specialists for pre-operative classification and surgeons. The need for such a system is obvious and is provided by the #Enzian classification. Future research is necessary to test its validity.

## Introduction

Since the very first description of endometriosis in 1860 (Rokitansky, 1860), several attempts have been made to classify and describe the anatomical extent of endometriosis and adhesions caused by it. The classification systems have changed over the years, but none has provided a clinically useful system that describes both peritoneal, ovarian, and deep endometriosis (DE). Due to the inadequacy of the existing systems, leading experts suggested that one should use a combination of the three most popular systems, i.e., the revised American Society for Reproductive Medicine (rASRM) classification, the Enzian classification and the endometriosis fertility index (EFI) (Johnson et al., 2017). This is not practical. A correct morphological-anatomical description of endometriosis is a conditio *sine qua non* for comparing the effect of different therapies of endometriosis and to describe the natural course of the disease. Therefore, one single terminology to describe the location and extent of endometriosis is needed, and this terminology should be the same for imaging experts and surgeons.

Today, ovarian endometriomas and DE can be detected by ultrasound or magnetic resonance imaging (MRI), (Guerriero et al., 2015a; Guerriero, et al., 2015b; Guerriero, et al., 2016; Gerges et al., 2021). Adhesions can also be suspected on transvaginal ultrasound (TVS) (Gerges et al., 2017; Holland et al., 2010). However, superficial peritoneal lesions cannot be reliably diagnosed by any imaging method (Kiesel and Sourouni, 2019). As a result, we now can counsel women appropriately before surgery; we can stage endometriosis, predict the complexity of surgery, the risk of surgical complications, and the likely outcome of treatment based on imaging results. The ideal classification system should be useful both for describing disease extent based on imaging results and based on surgical findings. Endometriosis should be managed by a multidisciplinary team, including radiologists, sonographers, gynaecologists, and experts in gynaecological, colorectal, and urological surgery. Therefore, a common language and classification system is needed.

## The rASRM score

**Figure 1:**
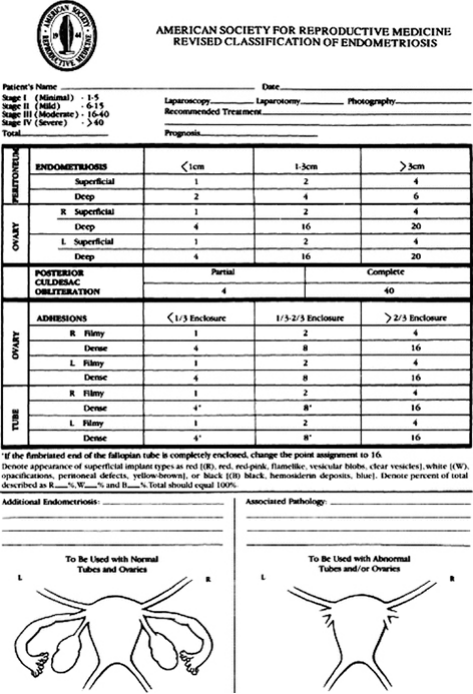
The rASRM score.

The American Fertility Society (AFS) first presented their classification system for endometriosis and pelvic adhesions in 1979 (The American Fertility Society, 1979). After revisions in 1985 (rAFS score) and 1996, this classification system is now known as the revised American Society for Reproductive Medicine (rASRM) classification (American Society for Reproductive Medicine, 1996). The rASRM classification divides endometriosis into four stages: minimal, mild, moderate, and severe (Figure 1). Changes involving the peritoneum, the fallopian tubes and ovaries are used to stage the disease. DE is not taken into account. When using the rASRM system, different points are assigned depending on whether the endometriotic lesion is deep or superficial, the size of the endometriotic lesion, and the type (filmy or dense) and extent of adhesions involving the fallopian tubes, ovaries, and the pouch of Douglas. The points are added to a total score, and the total score is used to stratify the disease into one of the four stages. The rASRM classification was originally designed to classify endometriosis via direct visualisation of the pelvic organs at laparoscopy or laparotomy. However, a diagnostic laparoscopy cannot determine the extent of the disease in the extraperitoneal space or in the pelvic organs themselves (bowel, bladder, uterus) without extensive excision. Diagnostic laparoscopy is unsuitable for complete description of the disease. The main advantage of the rASRM classification is its long-standing history and worldwide use. Over the past 40 years, scientific publications have mainly referred to the rASRM stage to describe the extent of endometriosis and compare the effect of different treatments for endometriosis (Keckstein and Hudelist, 2021). The rASRM classification focuses on the effects of endometriosis on fertility caused by peritoneal and ovarian implants and secondary adhesions. Its main disadvantage is that it does not cover the full spectrum of the disease; it does not describe extra-genital DE, such as DE involving bowel, bladder, rectovaginal septum or ureters, nor does it describe adenomyosis.

Attempts have been made to use TVS or MRI to stage endometriosis preoperatively using the rASRM classification. In a retrospective study including 204 women, Leonardi et al. (2020) found the accuracy of TVS for prediction of the surgical rASRM stage to be 53.4% for stage I, and 93.8%, 89.7% and 93.1% for stages II, III and IV, respectively. This shows that the performance of TVS was better for the higher disease stages. In a prospective study including 201 women, Holland et al. (2010) found good agreement between TVS findings and the surgical rASRM stage (absent, minimal, mild, moderate, and severe endometriosis; quadratic weighted kappa = 0.786). In a small cohort study including 65 patients Williams et al. (2020) demonstrated that kissing ovaries and retropositioned ovaries on pre-operative MRI were associated with high intra-operative rASRM stages of endometriosis.

Vercellini et al. (2007) analysed the association between the rASRM stage of endometriosis and the severity of different types of pelvic pain in over 1000 patients. In line with previous findings by Fedele at al. (1992) a correlation between the rASRM stage and the severity of pain was observed only for dysmenorrhoea and non-menstrual pain. This led to the conclusion that the association between the rASRM stage and the degree and type of pelvic pain symptoms was inconsistent. Chapron et al. (2003) were unable to find a correlation between rASRM stages and pain symptoms in women with DE. This is in line with a review by Andres and colleagues that showed poor correlation between the rASRM score and symptom severity (Andres et al., 2018). Poor correlation between the rASRM stage and natural pregnancy rates after endometriosis surgery has also been reported (Adamson, 2011; Vercellini et al., 2006).

Can the rASRM stage predict the complexity of surgery and surgical complications? In a retrospective study including 401 patients undergoing surgery for various stages of endometriosis, only women with rASRM stage 4 disease experienced Clavien-Dindo (CD) grade II and III complications (such as need for blood transfusion or surgical interventions) (Nicolaus et al., 2020). Similarly, in a study including 112 women with endometriosis undergoing hysterectomy, Ucella et al. (2016) found that surgical complications were more common in rASRM stage III-IV than stage I-II endometriosis. Poupon and colleagues reported no significant differences in major surgical complications between rASRM stages I-II and rASRM stages III-V (Poupon et al., 2019). However, both Clavien-Dindo complications grade I-II, grade III and voiding problems were more common in women with endometriosis rASRM stage III-IV than rASRM stage I-II (17% vs. 11%, 7% vs. 4%, and 7% vs. 3%). The results do suggest more complications in the higher stages.

## The EFI (Endometriosis Fertility Index)

**Figure 2:**
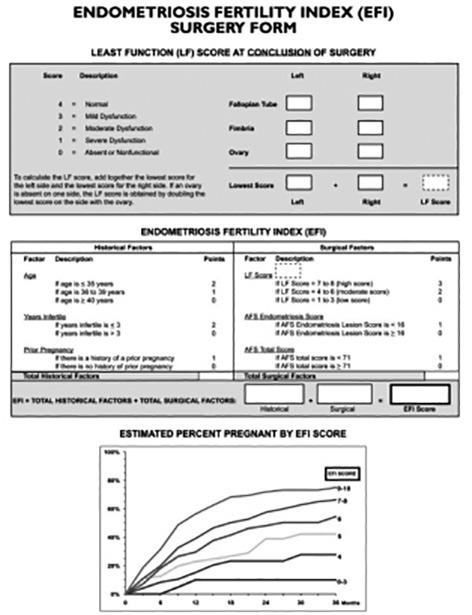
The EFI (Endometriosis Fertility Index).

In 2010, the Endometriosis Fertility Index (EFI) was suggested. It was designed by Adamson and Pasta (2010) to provide clinicians with a tool to predict the likelihood of natural conception occurring after surgery for endometriosis. The EFI was created using a comprehensive statistical analysis of prospectively collected data from a large number of infertility patients undergoing surgery for endometriosis. It is a 10-point scoring system, which includes variables such as patient characteristics (age, duration of infertility and history of prior pregnancy), the rASRM classification, and results of visual assessment of the fallopian tubes, tubal fimbriae, and ovaries during surgery. The tubo- ovarian function is estimated by assigning a “least- function score” (Figure 2). The EFI is assigned at the end of surgery. Endometriosis contributes a maximum of two points to the EFI score. Therefore, the name Endometriosis Fertility Index may be misleading.

Like the rASRM score, the EFI is primarily used to describe impaired fertility. Its clinical applicability and value to predict spontaneous pregnancy rates in endometriosis patients following endometriosis surgery has been confirmed in several studies (Boujenah et al., 2015; Garavaglia et al., 2015; Tomassetti et al., 2013; Tomassetti et al., 2021). Tubal function, reflected by the least function score, appears to be the key variable for prediction of fertility after surgery when one uses the EFI. The EFI has gained worldwide acceptance amongst reproductive surgeons and clinicians working with medically assisted reproduction. The World Endometriosis Society (WES) has supported its use to predict fertility after endometriosis surgery (Johnson et al., 2017) and the Consensus on Recording Deep Endometriosis Surgery (CORDES) statement suggests that EFI can be used to predict the probability of spontaneous conception after surgery for endometriosis (Vanhie et al., 2016).

Tomasetti et al. (2019) found that the EFI could be calculated based on clinical information and TVS results. This required evaluation of tubal patency with hystero-salpingo-contrast- sonography (HyCoSy). Adding information from a diagnostic laparoscopy improved agreement with the final EFI (end-of-surgery EFI score) by only 10% (absolute percentage). This was considered a limited improvement when weighed against the invasiveness of a surgical procedure. Future studies are needed to confirm these findings and to test their applicability in everyday clinical practice. A prerequisite for assigning an EFI score before surgery is expert ultrasonography.

## The Enzian and (#) Enzian classifications

Stimulated by the fact that the commonly used rASRM classification does not adequately describe DE, the Austrian-German-Swiss Scientific Endometriosis Foundation (Stiftung Endometrioseforschung/SEF) created and published the Enzian classification of endometriosis in 2003. The aim was to better describe and stage DE and to add missing information to the r-ASRM classification (Keckstein et al., 2003; Tuttlies et al., 2005). The Enzian classification was revised in 2009 to simplify its use. The rapid development of surgery for DE required a detailed description of the disease to enable comparison of the effects and risks of complications between different surgical techniques and of the accuracy between diagnostic methods. In contrast to the rASRM and EFI classifications, the Enzian classification describes DE involving the vagina, uterosacral ligaments, bladder, ureter, bowel, uterus, and other extragenital localisations. It also takes into account the size of the DE lesions. When using the Enzian classification, the pelvis is divided into three compartments. Compartment A includes the rectovaginal space, vagina, and torus uterinus (cranio-caudal axis), compartment B includes the utero-sacral ligaments, cardinal ligaments, parametrial space and pelvic sidewall (medio-lateral axis), and compartment C includes the rectum and sigmoid colon up to 16 cm from the anal verge (cranio-caudal axis but posterior to compartment A). The grade, i.e. the severity of endometriosis excluding minor peritoneal lesions with less than 5 mm infiltration depth, is defined for each of these three compartment as follows: grade 1 means infiltration <1 cm, grade 2 infiltration 1-3 cm, grade 3 infiltration >3 cm. Uterine involvement and other extragenital locations of DE (compartment F) are described as adenomyosis (FA), bladder DE (FB), extrinsic and/or intrinsic ureteric involvement with signs of ureteric obstruction (FU), bowel DE cranial to the rectosigmoid junction (FI) (>16 cm from the anal verge; upper sigmoid, transverse colon, caecum, appendix, small bowel), and other locations, e.g. the abdominal wall, diaphragm or nerve/sacral root involvement (FO).

Di Paola et al. (2015) and Burla et al. (2019) compared MRI findings with surgical findings using the Enzian classification and found good agreement between surgical and MRI Enzian classifications. Thomassin-Naggara et al. (2020) showed that the Enzian classification based on MRI findings is reproducible and correlates with surgical findings. Their retrospective observational study included 150 patients with DE that underwent MRI and subsequent surgery. The MRI based and surgical Enzian classifications were concordant for DE lesions in the A compartment in 78.7% (118/150), for B lesions in 34.7% (52/150) and for C lesions (colorectal DE) in 82.7% (124/150). Agreement between the radiologists assessing the MR images (inter-observer agreement) was good for DE in the A and C compartments but poor for lesions in the B compartment. Operating times and hospital stays were longer in patients with A2 than A0 lesions, B2 than B0 lesions, C3 than C2 lesions and C2 than C0 lesions according to MRI. This illustrates that there was an association between the Enzian disease grades assessed by MRI and surgical complexity. Patients with vaginal or rectosigmoid involvement (compartment A and C) according to MRI were six and three times more likely to experience grade III Clavien- Dindo complications (i.e., complications requiring surgical, endoscopic, or radiological intervention) than patients without vaginal or rectal DE.

Hudelist et al. (2019) evaluated the lesion location and size according to the Enzian classification by using preoperative TVS. They compared the ultrasound results with surgical findings in 195 women with DE and found good agreement between ultrasound and surgical findings especially for Enzian compartments A, C and FB. Concordance was highest for Enzian compartment C (rectosigmoid), in which 86% of all TVS C3 lesions were confirmed at surgery. Results were similar for Enzian compartment A (vagina, rectovaginal septum). In agreement with results of MRI studies, concordance between TVS findings and surgical findings was poorer for B lesions (uterosacral ligaments, parametria) with 71% of TVS B2 lesions being confirmed at surgery. In most cases of discordant findings, TVS underestimated lesion size by 1 severity grade compared with the intraoperative findings. TVS detected DE in compartments A, B, C, and FB with a sensitivity of 84%, 91%, 92%, and 88%, respectively, and specificity 85%, 73%, 95%, and 99%.

Two studies have examined the correlation between the Enzian classification of DE (location, grade) and the severity of preoperative pain symptoms. Both found a correlation (Haas et al., 2013; Montanari et al., 2019). Several groups have shown that operating times and risk of surgical complications can be predicted by the Enzian classification. Haas et al. (2013) demonstrated that intra-operative Enzian classification correlated with the duration of the surgical procedure. To create a prediction model for the risk of surgical complications, Poupon et al. (2019) developed a nomogram based on three simple criteria; the age of the patient, previous surgery for DE, and the extent of disease described by the surgical Enzian classification. Patients were classified as being at low, intermediate or high risk of surgical complications based on the Enzian classification (low risk defined as A0, A1, B1 and C0, intermediate risk as A2 and/or B2, high risk as A3, B3 or C1). The risk of surgical complications was lowest in the low-risk group and highest in the high-risk group. Imboden et al. (2021) reported a higher risk of postoperative voiding dysfunction in patients with DE involving the B compartment, especially for B3 lesions. Finally, Nicolaus et al. (2020) found a statistically significant 3.5-fold increased risk of Clavien-Dindo complications grade II or higher in the presence DE in the Enzian score. Furhermore, an Enzian C3 finding increased the risk of complications greater than Clavien- Dindo grade I 56.3 times (p < 0.001). Based on the above information, several guidelines suggest the use of the Enzian classification for desciption of endometriosis (Johnson et al., 2017; Ulrich et al., 2014; Ulrich et al., 2013; Vanhie et al., 2016).

**Figure 3:**
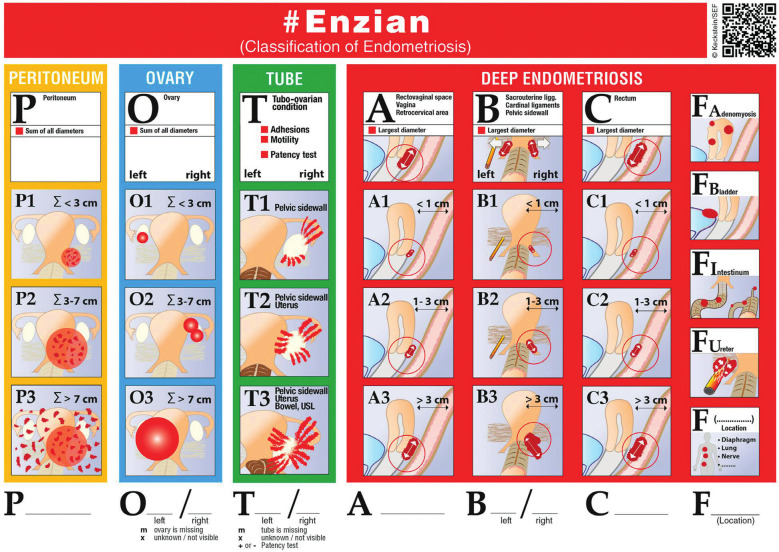
The #Enzian classification now also including peritoneal (“P”), and ovarian endometriosis (“O”) as well as adnexal adhesions (“T”).

A major criticism of the Enzian classification has been its focus on retroperitoneal, deep infiltrating disease. To overcome this, the #Enzian classification (Figure 3) was created. It is based on consensus between experts after discussions in 2019 and 2020 (Keckstein et al., 2021). The #Enzian classification includes description and classification of peritoneal and ovarian endometriosis and of tubal adhesions, tubal mobility and patency. The novelty of the #Enzian classification is that it takes all the structural manifestations of the disease into account and so allows a complete description of the disease. It can be used both for surgical staging and for staging using imaging, with the exception that superficial peritoneal disease is poorly detectable by current imaging methods and that MRI has limited ability to detect adhesions. The IDEA (International Deep Endometriosis Analysis group) terminology to describe the sonographic appearance of DE (Guerriero et al., 2016) can be incorporated into the #Enzian system. Future studies are needed to test the reproducibility of the #Enzian system when used by surgeons and imaging specialists, the diagnostic accuracy of MRI and TVS when using the #Enzian classification for presurgical staging, and the association of the #Enzian classification with symptoms (including subfertility) and surgical complications.

## Summary and conclusions

A comparison between the rASRM, EFI, Enzian and #Enzian classification systems is provided in Table I. The developments in imaging in the last two decades have not only improved non- invasive diagnosis of endometriosis but also opened a possibility to describe the extent of the disease before surgery (“pre-operative staging”). Although the rASRM classification is the most frequently used system for describing the severity of endometriosis, it does not take into account the retroperitoneal, deep infiltrating phenotypes of endometriosis. The EFI has been extensively validated regarding prediction of pregnancy rates after surgery for endometriosis and appears to be a suitable tool for this purpose. However, like the rASRM, the EFI does not describe DE. Because neither the EFI nor the rASRM classification takes DE into account, they do not reflect the full spectrum of the disease. Therefore, they are not suited to predict the complexity of surgery and risk of surgical complications. Description of the full spectrum of the disease is also needed if one wants to investigate the association between different types of endometriosis and symptoms or the effect of treatment on different types of endometriosis.

**Table I:** Comparison of the different classification systems, rASRM, EFI and Enzian including its recently updated and proposed version #Enzian. The advantages and disadvantages of the systems are compared (- not suitable, + little, ++ moderately and +++ well suitable, u.i., under investigation).

	r-ASRM	EFI	Enzian	#Enzian
Clinical acceptance	+++	++	++	u.i.
Applicability with non-invasive methods (TVS/MRI)	++	+	++	u.i.
Defining deep endometriosis (DE)	+	+	+++	u.i.
Correlation with surgical complexity/complications rates	++	-	+++	u.i.
Correlation with infertility	+++	+++	u.i.	u.i.
Correlation with surgical complexity, complication rates	++	-	+++	u.i.
Correlation with pain symptoms	-	-	+++	u.i.

Until recently, the Enzian classification was predominantly used to describe DE. It is a commonly used method of staging DE in internationally recognised centres of expertise (Vanhie et al., 2016). A great advantage of the Enzian classification is that it can be used to describe the disease on ultrasound or MRI. However, when using ultrasound or MRI the are a few problems. First, MRI measurements of the size of DE affecting the B compartment (uterosacral ligaments, parametrium) are poorly reproducible, and secondly MRI findings of lesions in the B-compartment show poor concordance with surgical findings. Similarly, measurements of parametrial DE by TVS, which have been investigated in only one study (Hudelist et al., 2021) are less reliable (when compared with surgical findings) than measurements of lesions in the A, C and FB compartments. In favour of the Enzian system, there is increasing evidence that the Enzian classification can predict surgical complexity and complications. The #Enzian classification is a step towards the use of one universal classification system, because it includes description not only of DE but also of peritoneal and ovarian endometriosis, adnexal adhesions, and tubal patency. To what extent the #Enzian classification can be used to predict fertility needs to be investigated. We also need studies examining the association between the #Enzian classification and symptoms and between the #Enzian classification and surgical complexity. The inter- and intra-observer reproducibility of the classification when used by surgeons and imaging experts also needs to be investigated.

The ideal classification system for endometriosis should describe the sites and extent of the disease, be related to surgical complexity and to disease- associated symptoms, including subfertility. Most importantly, it should be possible to use the classification system both by imaging specialists for pre-operative classification and by surgeons. The need for such a system is obvious and is provided by the #Enzian classification. The validation of this system regarding the above aspects should be the focus of future research.
